# Clinical implication of smartphone‐based atrial fibrillation detection system

**DOI:** 10.1002/joa3.13172

**Published:** 2024-10-21

**Authors:** Naoya Kataoka, Teruhiko Imamura

**Affiliations:** ^1^ Second Department of Internal Medicine University of Toyama Toyama Japan

To Editor:

Early detection of asymptomatic atrial fibrillation (AF) and the development of effective management strategies remain global concerns. The authors investigated the prevalence of AF in the Indonesian population using the AliveCor Kardia™ Mobile system, identifying a 3.2% prevalence of AF among visitors to referral hospitals based on smartphone‐based diagnoses in Indonesia.^1^ However, several important issues warrant consideration.

The duration of AF measurement using the AliveCor™ system in the authors' study is not clearly specified, which is a critical factor as it directly influences the AF detection rate.^1^ Furthermore, it is unclear whether the authors differentiated between types of AF, such as persistent AF and paroxysmal AF, which carry distinct clinical implications. A significant proportion of the detected cases may represent persistent AF or symptomatic paroxysmal AF.

A notable limitation of the study is the transient nature of the AF measurements. Devices like AliveCor™, which offer only temporary monitoring, are less likely to detect asymptomatic paroxysmal AF, a crucial therapeutic target. In contrast, wearable devices, which provide continuous monitoring, may be more effective in identifying such cases.^2^

Current guidelines define AF based on a 12‐lead electrocardiogram or 24‐h Holter monitoring. Sub‐clinical AF, which may be detected by easy‐to‐use devices like AliveCor™, is not typically classified as AF according to these guidelines. Additionally, recent studies have shown that AF episodes lasting longer than 24 h are significantly associated with an increased risk of thromboembolic events, whereas transient subclinical AF may not carry the same risk.^3^

## CONFLICT OF INTEREST STATEMENT

The authors declare no conflicts of interest.
